# Pedigree analysis in the mhorr gazelle (*Nanger dama mhorr*): Genetic variability evolution of the captive population

**DOI:** 10.1002/ece3.10876

**Published:** 2024-02-17

**Authors:** Sonia Domínguez, Isabel Cervantes, Juan Pablo Gutiérrez, Eulalia Moreno

**Affiliations:** ^1^ Estación Experimental de Zonas Áridas‐CSIC Almería Spain; ^2^ Departamento de Producción Animal, Facultad de Veterinaria UCM Madrid Spain

**Keywords:** captive breeding program, critically endangered, dama gazelle, genetic diversity, mating strategy, pedigree analysis

## Abstract

Breeding programs have an essential role in the recovery of threatened populations through optimal genetic management and mating strategies. The dama gazelle (*Nanger dama*) is a North African ungulate listed as critically endangered. The *mhorr* subspecies is extinct in the wild and currently survives thanks to the creation in 1971 of an ex situ breeding program. The aim of the present study was to assess the evolution of genetic variability in this mhorr gazelle captive population, as well as the mating strategy used in two reference populations studied (Almeria and Europe). The entire pedigree, with 2739 animals, was analyzed to measure demographic characters, pedigree completeness level, probability of gene origin, level of relatedness and genetic structure of the population. The population size has been progressively increasing, with up to 264 individuals alive in Europe at the time of the study. The average number of equivalent complete generations was 5.55. The effective number of founders and ancestors was both 3, and the founder genome equivalent was 1.99. The genetic contributions of the four main ancestors were unbalanced. The average values of inbreeding and average relatedness for the whole pedigree were, respectively, 28.34% and 50.14%. The effective population size was 8.7 by individual increase in inbreeding and 9.8 by individual increase in coancestry. *F*‐statistics evidenced a very small level of population subdivision (*F*_ST_ = 0.033370). The mating strategy used, based on the minimum coancestry of the individuals, has minimized the losses of genetic variability and helped to balance the genetic contributions between ancestors. The strategy also avoided large subdivisions within the population and the appearance of new bottlenecks. This study shows how pedigree analysis can both be used to determine the genetic variability of the population and to assess the influence of the mating strategy used in the breeding program on such variability.

## Graphical abstract

This paper describes the genetic characterization of the mhorr gazelle population in captivity obtained through pedigree analysis. The genetic variability evolution shows how the mating strategy used has allowed maximizing the genetic potential of the population despite the low number of founders. The study of two living reference populations has made it possible to know the level of current genetic variability.

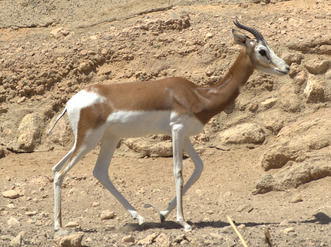

## 1 INTRODUCTION

Maintaining genetic diversity is one of the most important requirements for the conservation of biodiversity (Frankham, 1995). Ex situ captive breeding programs were created as intensive population management plans for threatened species with the aim of retaining as much genetic diversity of these populations as possible (Ballou et al., 2010). Relationships among family members within an ex situ breeding program are recorded in pedigrees or studbooks (Van Dyke, 2008). This genealogical information can be used to characterize the genetic diversity and gene flow of populations through a pedigree analysis, where genetic parameters related to the level of inbreeding and population structure are obtained (Lozada et al., 2023). Furthermore, long‐term pedigree data provide valuable insight into the evolutionary dynamics of populations, including studies of inbreeding depression, genetic architecture of quantitative traits, selection effects and inbreeding avoidance (Kruurk & Hill, 2008). It should be noted that these analyses based on pedigree data depend very much on the completeness of the recorded information, so accuracy on registering historical events is crucial for reliable results (Siderits et al., 2013). In combination with the study of demographic parameters, the analysis of genealogical information enables designing and implementing optimal breeding plans to maintain an adequate genetic pool within the population (Valera et al., 2005).

Genealogical information belonging to the ex situ population of mhorr gazelle (*Nanger dama mhorr*) has been recorded for over 50 years, which provides an excellent opportunity to investigate the genetic characteristics of this population and define a long‐term genetic management plan, including the selection of animals for the restoration of wild populations. The main aim of the present study is to establish, for the first time, the evolution across generations of both, the genetic variability and the genetic structure of the captive population of mhorr gazelle, by analyzing the whole pedigree information included in its studbook since 1971. We also compared results obtained by using different methodological approaches (demographic versus pedigree) for a relevant indicator of genetic variability such as the effective population size. Results of this study helped us to evaluate the adequateness of the mating strategies used in the captive breeding program over time.

## 2 MATERIALS AND METHODS

### 2.1 Study species and studied populations

**FIGURE 1:**
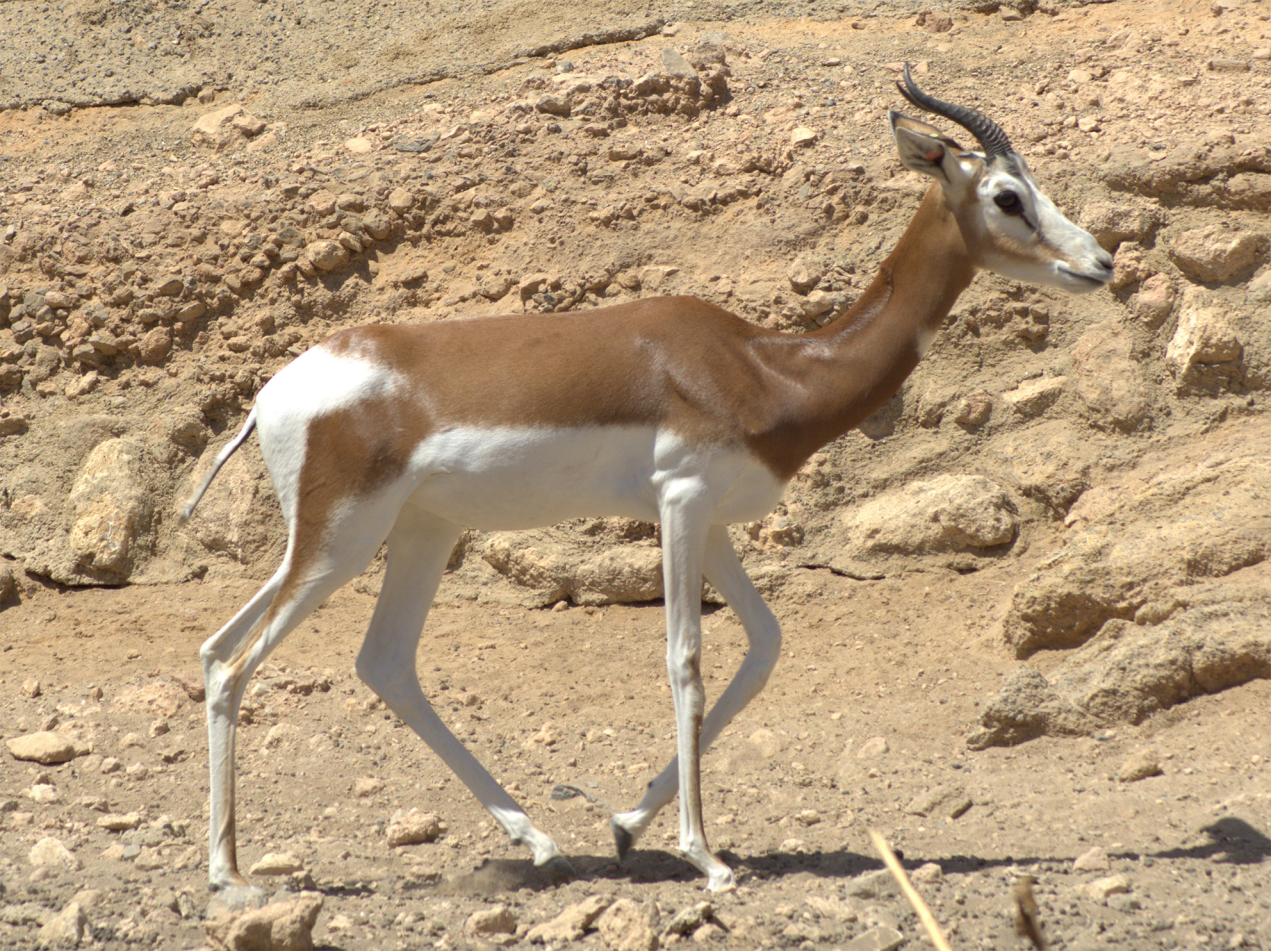
Female of mhorr gazelle at the Estación Experimental de Zonas Áridas (Almeria, Spain).

The dama gazelle (*Nanger dama*) is a North African ungulate listed as critically endangered in the Red List of Threatened Species of the International Union for Conservation of Nature (IUCN, 2016). The main causes of its decline have been indiscriminate hunting and habitat loss (Durant et al., 2014). Dama gazelle (Figure 1) is a large‐size and sexually dimorphic gazelle, with individuals weighing from 40 to 75 kg. Females reach sexual maturity at 9–12 months and males at 18–24 months. Single births follow a 6.5 months gestation (Barbosa & Espeso, 2005). Three subspecies are currently recognized: *Nanger dama mhorr*, *Nanger dama dama* and *Nanger dama rufficollis* (Cano, 1984). No animal of *mhorr* subspecies has been seen in the wild since 1968 (Valverde, 2004), but it survives because of an ex situ captive breeding program established at the Estación Experimental de Zonas Áridas (Almeria, Spain) in 1971. The captive population expanded as a result of the participation of new zoological institutions throughout Europe, North America, Africa and the United Arab Emirates (Appendix S1).

In this study, we use mhorr gazelle pedigree information recorded in its studbook since the foundation of the captive breeding program in 1971 to December 2021. A total of 2739 animals (1363 males, 1325 females and 51 of unknown sex) were included in our pedigree analysis (whole pedigree, WP). In addition to these calculations made with the historical records of the entire pedigree, they were also analyzed for two reference populations that included only living animals: a population of 107 living individuals in Almeria (reference population of Almeria, RPA) and the other with 157 living individuals from 26 European zoos (reference population of Europe, RPE). The living animals included in these two reference populations are those currently managed within the EAZA Ex‐situ Programme (EEP), the conservation program for this species kept in European Association of Zoos and Aquaria (EAZA) institutions. Within the mhorr gazelle EEP, animals are regularly moved from one institution to another to optimize mating and minimize inbreeding. Breeding males are selected based on the minimum coancestry with the females to mate with. In the founding institution of Almeria, these breeding males are mostly replaced every year, while in other zoological institutions the same male generally remains for several years or even its entire life with the same group of females. Therefore, the purpose of establishing these two reference populations separately was to determine whether there were differences in genetic variability between them because of the mating management carried out within the captive breeding program.

### 2.2 Analyses performed

The following demographic parameters of interest were assessed:

- *Record frequency*: animals registered in the studbook per year. This frequency can be associated with the number of animals born during each period, since all individuals are registered at the time of birth, whether or not they become breeders in the future.
- *Average offspring per breeding animal*: the mean number of descendants was calculated from the animals with offspring registered in the studbook.
- *Generation intervals*: defined as the average age of parents at the birth of their progeny used for reproduction (James, 1977). This parameter was computed for the four pathways (father‐son, father‐daughter, mother‐son and mother‐daughter) using birth dates of registered animals together with those of their sires and dams.

The pedigree completeness was characterized using the following parameters:

- *Proportion of known ancestors per generation*: computed for each individual by calculating the proportion of ancestors known in each ascending generation (Boichard et al., 1997).
- *Number of equivalents complete generations* (*g*_e_): defined as the sum of the proportion of known ancestors over all traced generations (Maignel et al., 1996).

The genetic representation of the founder population was assessed by computing:

- *Effective number of founders* (ƒ_e_): defined as the number of equally contributing founders that would be expected to produce the same genetic diversity as in the population under study (Lacy, 1989). This was estimated following James (1972).
- *Effective number of ancestors* (ƒ_a_): is the minimum number of ancestors (founders or not) necessary to explain the complete genetic diversity of the population under study (Boichard et al., 1997).
- *Number of founder genome equivalents* (ƒ_g_): is the theoretically expected number of founders that would be required to provide the level of genetic diversity observed in the living population if the founders were all equally represented and had lost no alleles (Ballou & Lacy, 1995). This parameter was obtained following Caballero and Toro et al. (2000).

Subsequently, to characterize the level of inbreeding and relatedness in the population, the following parameters were calculated:

- *Individual inbreeding coefficient* (*F*): this is the probability that an individual has two identical alleles by descent (Wright, 1923) and was computed following Meuwissen and Luo (1992).
- *Partial inbreeding coefficients*: measure the probability than an individual is homozygous (identical by descent) for an allele descended from the specified founder. The sum, across all founders, of the partial inbreeding coefficients for a descendant is equal to the overall inbreeding coefficient for that individual (Lacy et al., 1996).
- *Average relatedness coefficient* (AR) *of each individual*: could be defined as twice the probability that two random alleles, one from the animal and the other from the population in the pedigree (including the animal), are identical by descendant (Dunner et al., 1998). This parameter was calculated with an algorithm equivalent to the one used by Colleau (2002) when the whole population is considered as a single group.
- *Effective population size* (*N*_e_): is defined as the number of breeding animals that would lead to the actual increase in inbreeding if they contribute equally to the next generation (Wright, 1931). Different methods were carried out to compute *N*_e_. Two of them used demographic information: sex ratio (Wright, 1931) and variance of family size (Hill, 1979). And another two methods used pedigree data: by individual increase in inbreeding (Gutierrez et al., 2009; Gutiérrez et al., 2008) and by individual increase in coancestry (Cervantes et al., 2011).

Finally, the study of the population structure of the mhorr gazelle was completed by calculating:

- *F‐statistics*: computed from genealogical information for each defined subpopulation (Wright, 1978). Wright's *F*‐statistics include *F*_IT_, *F*_ST_ and *F*_IS_ as measures of heterozygosity at various levels of population structure (Falconer & Mackay, 1996). These parameters were computed following Caballero and Toro (Caballero & Toro, 2000, 2002).

All these parameters above were computed for the WP, the RPA and the RPE, with the exception of the effective population size by demographic methods, which were only calculated for the RPA, as it is the population with the most complete genealogical information. For the calculation of the WP parameters, 302 of the 2739 animals in the studbook had no genealogy information on record. These animals belonged to two non‐European institutions: Al Ain Zoo (in the United Arab Emirates) with 293 animal records and Jardin Zoologique National de Rabat (in Morocco) with nine animal records. Previous analyses showed that including these animals without pedigree information in the calculations underestimated the inbreeding parameters of the overall population. Consequently, animals from these two institutions were only included in the computation of demographic parameters and pedigree completeness level, and they were excluded from the calculation of the rest of the genetic parameters. Therefore, for the analysis of demographic and pedigree completeness parameters a total of 2739 animal records were used, and for the remaining parameters, a total of 2437 animals were included. Finally, Wright's *F*‐statistics were computed just for the living population of Europe, whose genetic management is carried out jointly through the EEP of the mhorr gazelle (Table 1).

**TABLE 1:** Location and demographic data of the 27 active European institutions with mhorr gazelles (updated on 31 December 2021).

Institution name	Country	Total	Males	Females	Undetermined	Alive	Dead
Schoenbrunner Tiergarten	Austria	16	9	6	1	7	9
Zoo Planckendael	Belgium	8	8	0	0	8	0
Animal Park Auvergne	France	5	4	1	0	2	3
Bioparc de Doué‐la‐Fontaine	France	18	6	12	0	11	7
Parc Zoologique de Paris	France	6	0	6	0	4	2
Safari Parc du Haut Vivarais	France	13	13	0	0	6	7
Zoo de Montpellier	France	39	19	19	1	5	34
Münchner Tierpark Hellabrunn	Germany	171	78	89	4	14	157
Tierpark Berlin	Germany	104	39	65	0	6	98
Zoo Frankfurt	Germany	108	52	49	7	2	106
Budapest Zoo and Botanical Garden	Hungary	23	7	14	2	3	20
Bioparco di Roma	Italy	5	5	0	0	3	2
Zoom Torino	Italy	18	9	9	0	6	12
Zoosafari di Fasano	Italy	10	10	0	0	7	3
Rotterdam Zoo	Netherlands	40	16	23	1	5	35
Zoo Zamosc	Poland	5	5	0	0	3	2
Zoo Santo Inácio	Portugal	16	6	9	1	8	8
Bioparc Valencia	Spain	11	5	6	0	4	7
Estación Experimental de Zonas Áridas	Spain	985	497	487	1	107	878
La Reserva del Castillo de las Guardas	Spain	14	14	0	0	1	13
Marcelle Natureza	Spain	9	9	0	0	3	6
Oasys Parque Temático de Tabernas	Spain	28	25	3	0	8	20
Río Safari Elche	Spain	8	8	0	0	8	0
Zoo Aquarium de Madrid	Spain	37	21	16	0	9	28
Zoobotánico Jerez	Spain	67	35	30	2	7	60
Zoo de Barcelona	Spain	52	18	27	7	12	40
Kolmarden Wildlife Park	Sweden	5	1	4	0	5	0
	Total	1821	919	875	27	264	1557

All the analysis were performed using the ENDOG programme v4.8 (Gutiérrez & Goyache, 2005).

## 3 RESULTS

### 3.1 Demographic analyses

**FIGURE 2:**
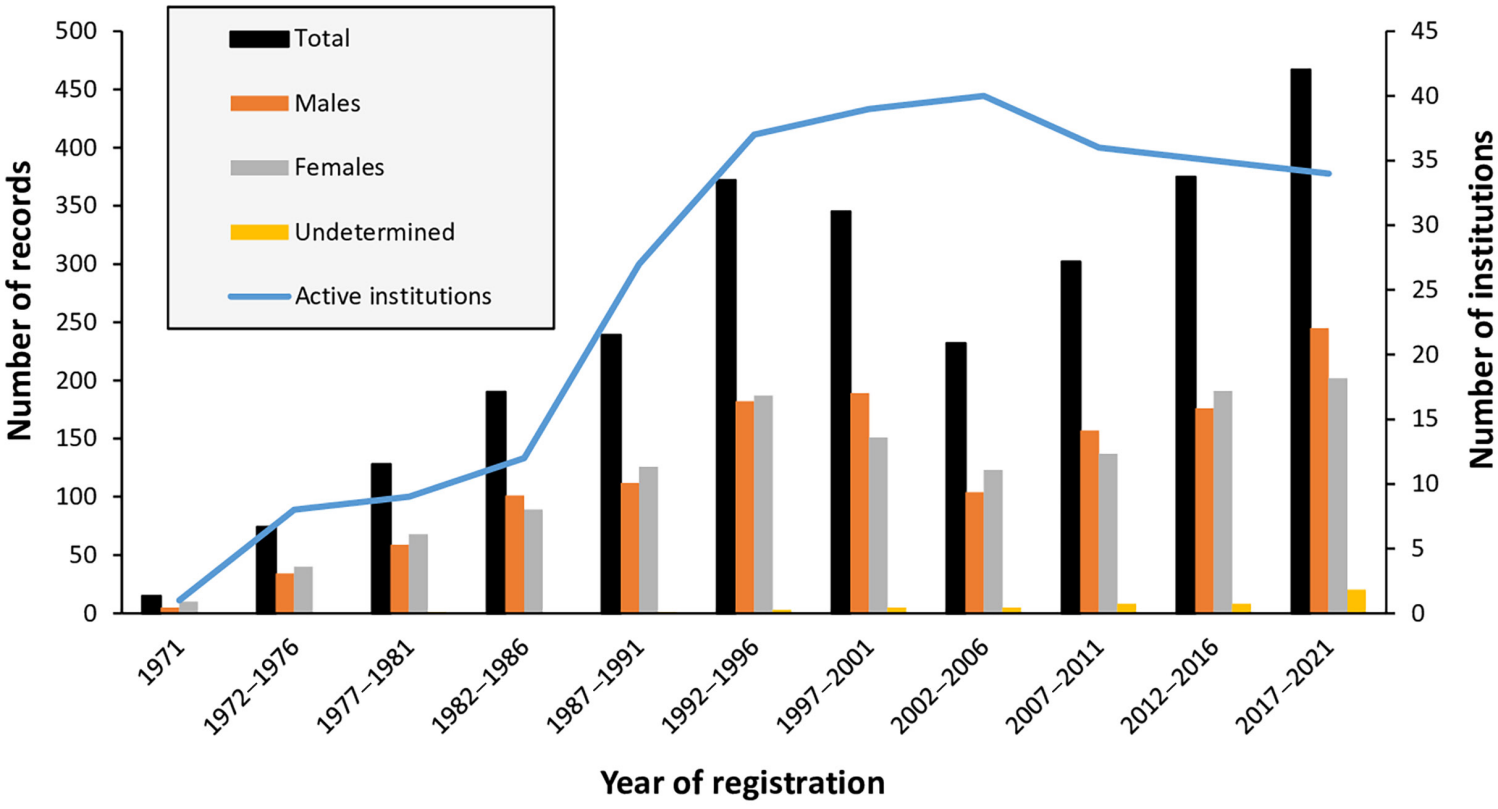
Number of animal records and active institutions in the mhorr gazelle studbook by periods of 5 years.

The number of animal records and participating institutions in the ex situ conservation program can be seen in Figure 2. Only in two consecutive periods (1997–2001 and 2002–2006) the number of records decreased, indicating a lower number of births during those years.

**FIGURE 3:**
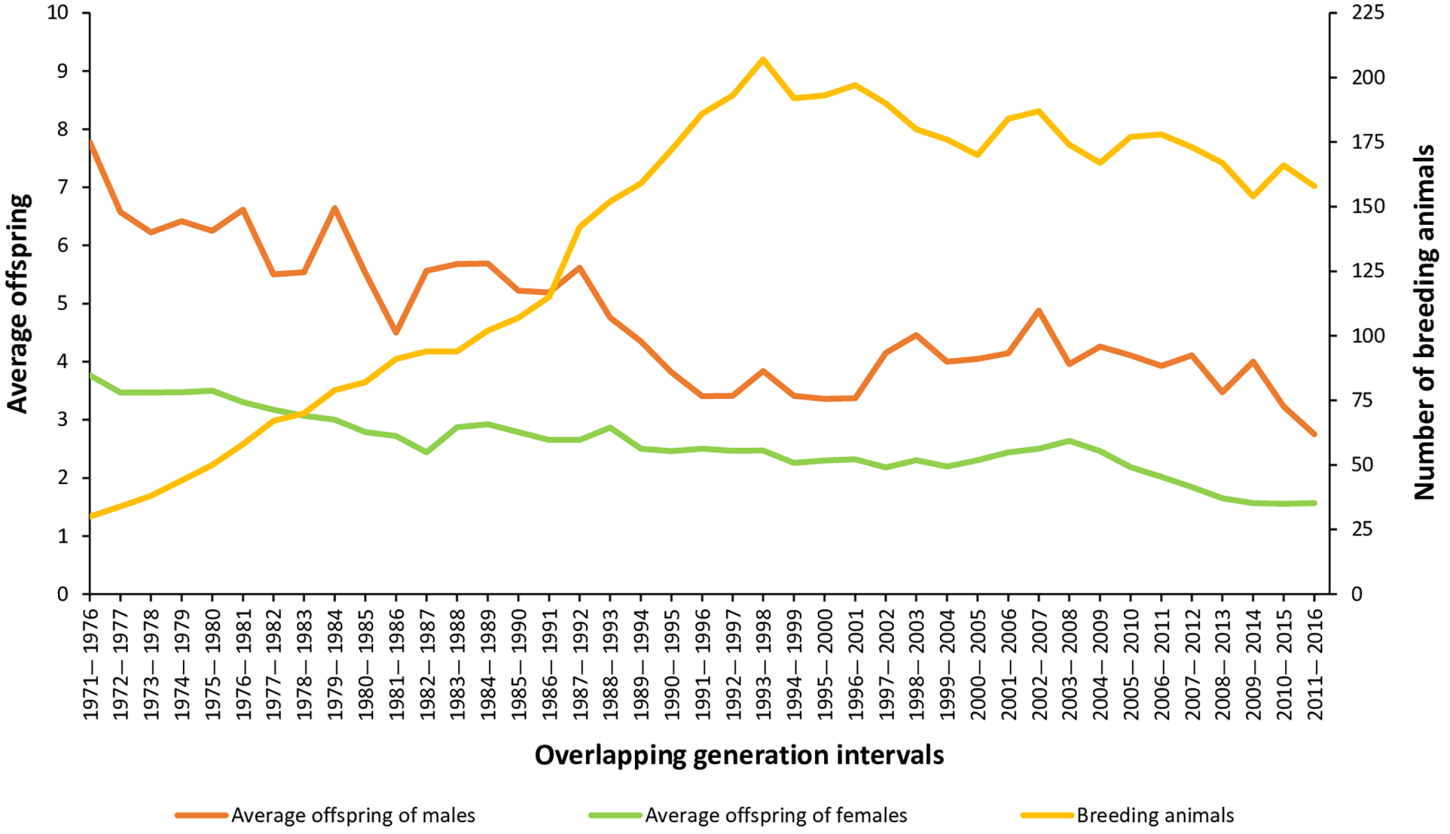
Average number of breeding animals and average number of offspring of males and females per generation interval registered in the mhorr gazelle studbook.

The proportion of animals with offspring registered in the studbook was 16.95% for males and 41.81% for females. This difference between both sexes was also maintained when only animals reaching sexual maturity were considered: 40.74% of the males and 69.77% of the females that reached sexual maturity had descendants. This result was significant (proportion test: *z* = 10.71; *p* < .01). Otherwise, the average number of offspring per breeding animal has been decreasing over generation intervals for both sexes due to growth in population size and, consequently, due to an increase in the number of animals available for reproduction (Figure 3).

The average generation interval for the entire pedigree was 5.85 years. This estimate was 5.87 years in the RPA and 5.76 years in the RPE. Table 2 shows the generation lengths calculated for the four pathways parent‐offspring.

**TABLE 2:** Generation intervals, standard deviations and standard errors for the four pathways parent‐offspring in the whole pedigree of the mhorr gazelle (WP), the reference population of Almeria (RPA) and the reference population of Europe (RPE).

Pathway	Whole pedigree				Almeria				Europe			
	*N*	Years	SD	SE	*N*	Years	SD	SE	*N*	Years	SD	SE
Sire‐son	214	5.96	2.66	±0.18	2	8.05	5.43	±3.84	9	5.72	3.27	±1.09
Sire‐daughter	503	5.89	2.59	±0.12	53	6.44	2.97	±0.41	44	6.13	2.53	±0.38
Dam‐son	210	5.72	3.09	±0.21	2	2.86	0.33	±0.23	9	4.38	2.20	±0.73
Dam‐daughter	492	5.81	2.88	±0.13	53	5.33	2.75	±0.38	44	5.69	2.83	±0.43

### 3.2 Pedigree completeness level

**FIGURE 4:**
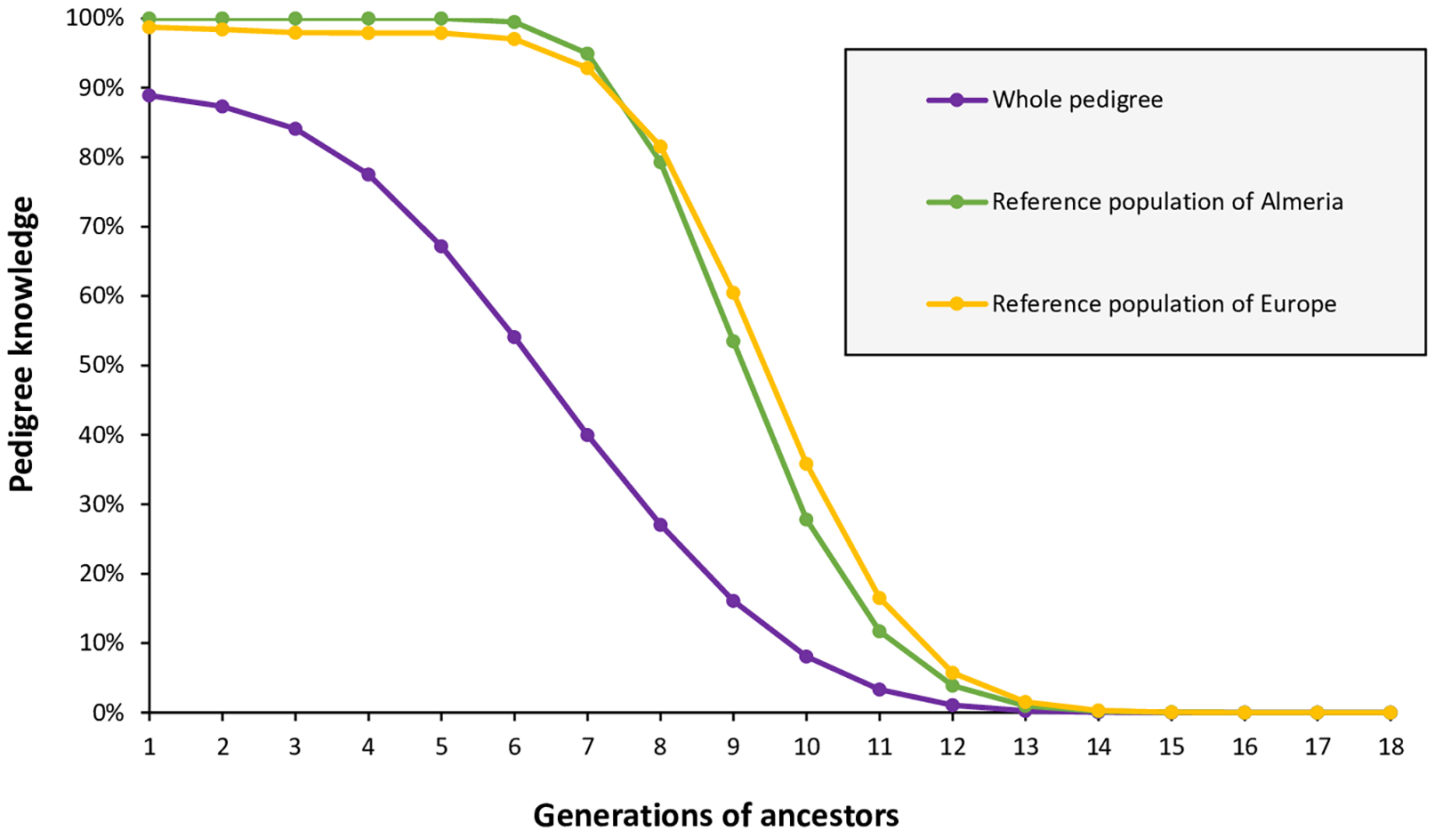
Completeness level of the mhorr gazelle studbook assessed by the average percentage of known ancestors per generation. Generation number 1 corresponds to parents, generation number 2 corresponds to grandparents, and so on. Information is provided for the whole pedigree (WP), the reference population of Almeria (RPA) and the reference population of Europe (RPE).

The amount of the available pedigree information for the WP, the RPA and the RPE was compared (Figure 4). For the first three generations of the WP, the percentage of known ancestors was higher than 80%. Subsequently, there was a significant decrease until the pedigree knowledge drops below 10% after the tenth generation. When only live animals from the RPA and the RPE were considered, the completeness of the genealogical information was 100% in the first five generations for the RPA and only slightly lower for RPE. In both cases, this percentage did not drop below 10% until the twelfth generation.

The number of equivalent complete generations was 5.55 for the WP, 8.72 for the RPA and 8.83 for the RPE.

### 3.3 Probability of gene origin

The parameters characterizing the genetic variability of the WP, the RPA and the RPE in terms of gene origin were also compared (Table 3). For the WP, eight ancestors were identified according to criteria by Boichard et al. (1997), but only four of them (one male and three females) explained more than 99% of the WP genetic variability. These four animals are among the first specimens that arrived in Almeria from Africa in 1971. The other four ancestors together do not reach 1% of the genetic variability. Regarding the RPA and the RPE, the same four ancestors were identified, but in this case, they did explain 100% of the genetic variability of each population. The contributions of these four ancestors identified for the whole as well as for both reference populations were clearly unbalanced (Table 4).

**TABLE 3:** Probability of gene origin parameters in the mhorr gazelle population, for the whole pedigree and the two reference populations (Almeria and Europe).

	Whole pedigree	Almeria	Europe
Total number of animals in the population	2437	107	157
Number of founders	9	4	8
Number of ancestors	8	4	4
Effective number of founders (ƒ_e_)	3	3	3
Effective number of ancestors (ƒ_a_)	3	3	3
Number of founder genome equivalents (ƒ_g_)	1.99	1.64	1.69

**TABLE 4:** Genetic contributions of the four main ancestors to the genetic variability of the whole pedigree (WP), the reference population of Almeria (RPA) and the reference population of Europe (RPE) of the mhorr gazelle.

Studbook number	Sex	Genetic contribution (%)		
		Whole pedigree	Almeria	Europe
1	Male	80.58	82.44	80.38
3	Female	2.81	3.99	4.19
4	Female	13.87	9.72	12.26
20	Female	2.74	3.85	3.17

### 3.4 Level of inbreeding and effective population size

The average values of *F* and AR for the WP were, respectively, 28.34% and 50.14%. The same values for the RPA were 28.32% and 45.13%, and for the RPE, 28.54% and 45.07%.

**FIGURE 5:**
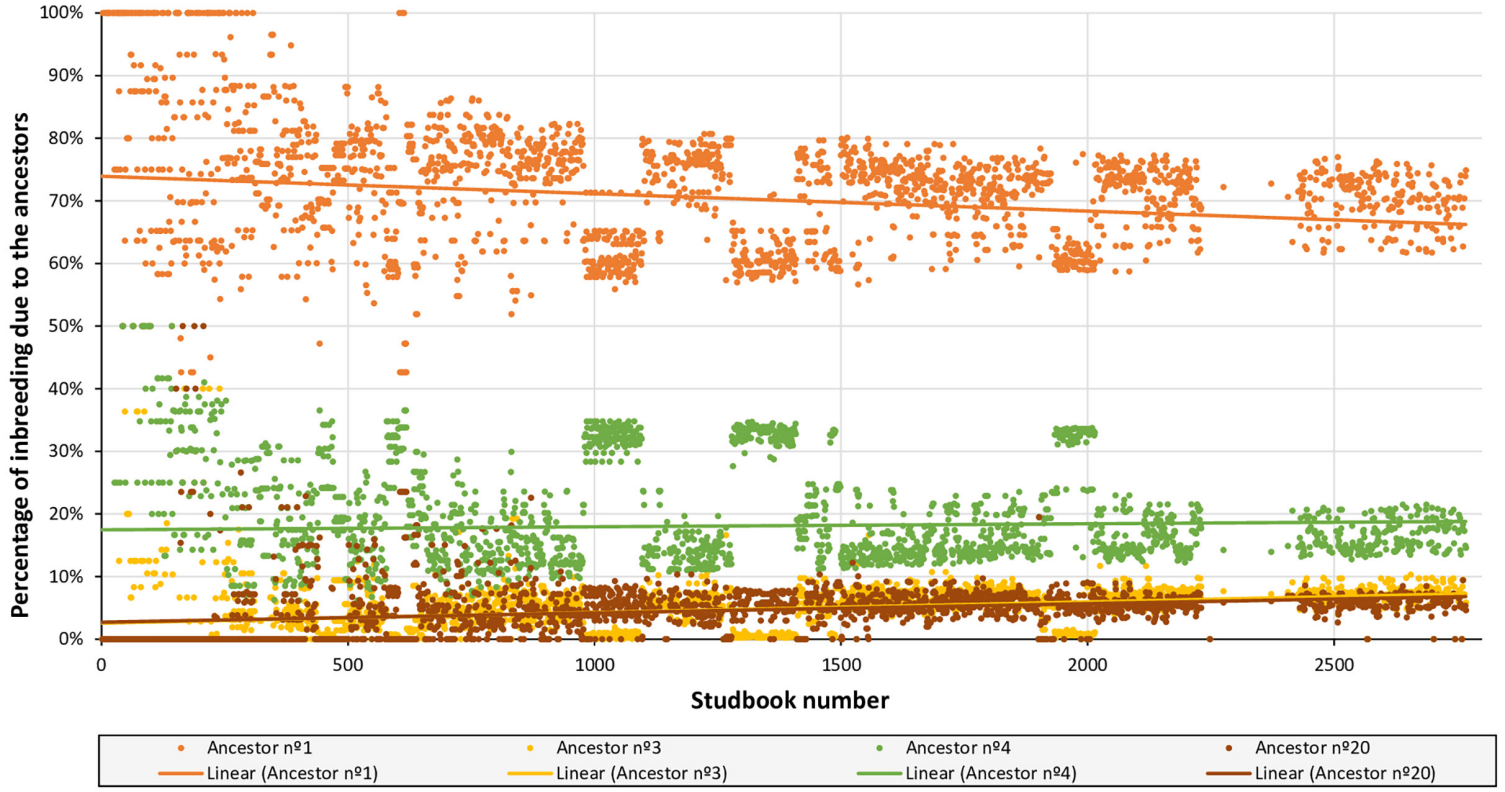
Percentage of inbreeding represented by the main four ancestors in each animal of the studbook and their trend lines (overlapped for ancestors 3 and 20).

Based on the partial inbreeding coefficients, the percentage of inbreeding of the animals in the studbook due to each of the four main ancestors was also calculated (Figure 5). It is worth noting how the partial inbreeding coefficients are those expected according to the genetic contribution of each ancestor. Likewise, the percentage of inbreeding due to ancestor number 1 was the one that contributed the most to the overall inbreeding of the individuals. It can also be seen that the percentage of inbreeding due to ancestor number 1 has decreased over time, and the percentage due to the ancestors 3, 4 and 20 has increased.

**FIGURE 6:**
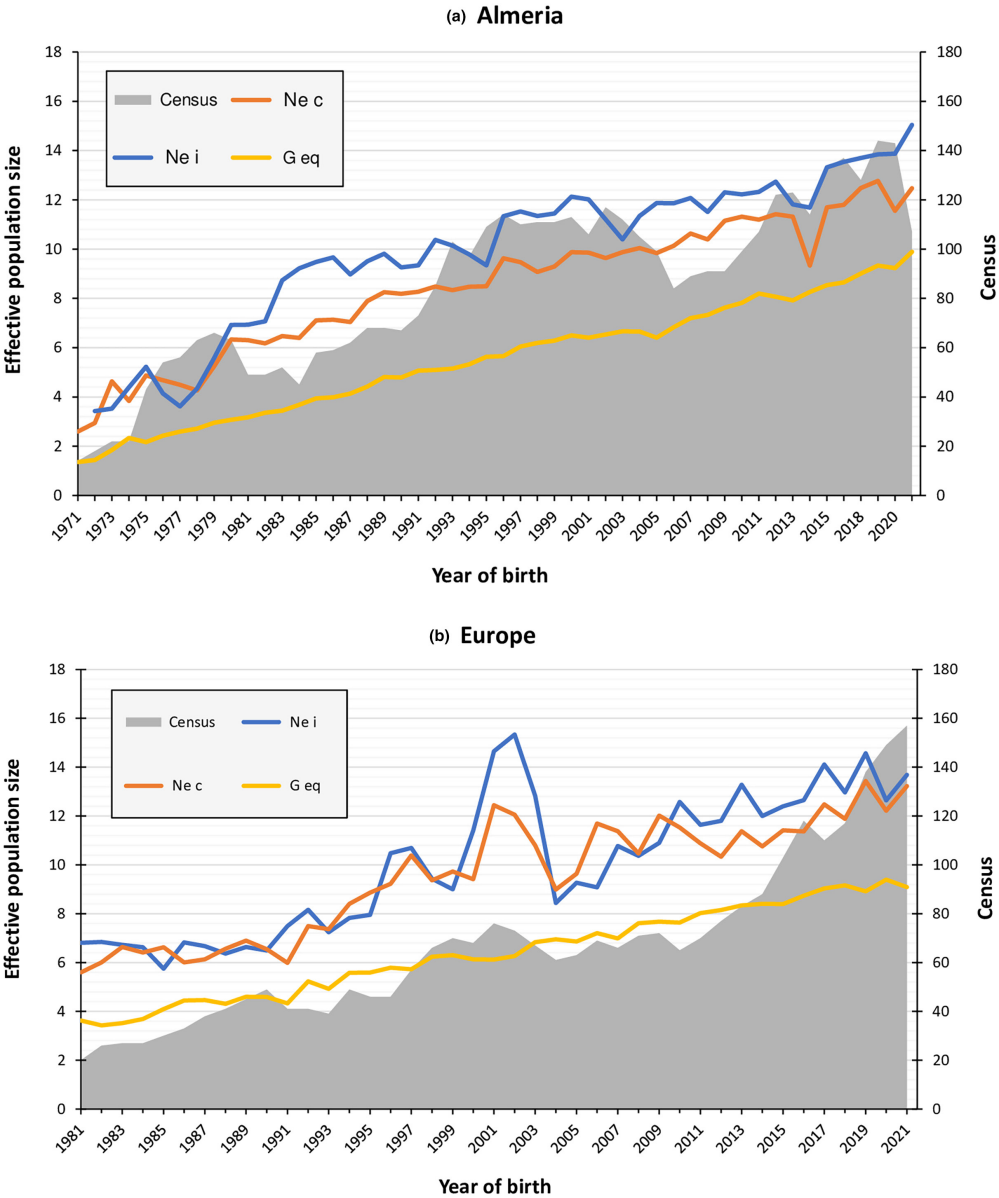
Evolution of the census, number of equivalent complete generations (*G*_eq_), effective population size based on individual increase in inbreeding (*N*_ei_) and effective population size based on individual increase in coancestry (*N*_ec_), in the reference populations of Almeria (a) and Europe (b).

The effective population size obtained by individual increase in inbreeding ($\bar{N_{\mathrm{ei}}}$) and by individual increase in coancestry ($\bar{N_{\mathrm{ec}}}$) for the WP were, respectively, 8.7 and 9.8. The same analyses for the RPA were 13.2 and 12.1, and for the RPE, 13.4 and 12.8. The values obtained for the WP were lower than those obtained for the reference populations, probably because it includes historical populations located in North America with more closely related animals. The evolution of $\bar{N_{\mathrm{ei}}}$ and $\bar{N_{\mathrm{ec}}}$ over time in the two reference populations was also assessed (Figure 6a,b). In Almeria, the value of $\bar{N_{\mathrm{ei}}}$ remained higher than $\bar{N_{\mathrm{ec}}}$ from 1978 to the present, whereas in Europe this only happened from 2010. It should be noted that the population of Europe was founded in 1981, 10 years later than the Almeria population. In addition, the ratio $\bar{N_{\mathrm{ec}}}/\bar{N_{\mathrm{ei}}}$ was calculated to analyze the structure of the populations (Cervantes et al., 2011). The result obtained corresponds to the number of equivalent subpopulations that constitute the population. Therefore, in a population without subdivision, the result would be 1. The ratio obtained was 1.12 for the WP, 0.92 for the RPA and 0.96 for the RPE. When the ratio was calculated for the two reference populations together, the result was 1.04.

**FIGURE 7:**
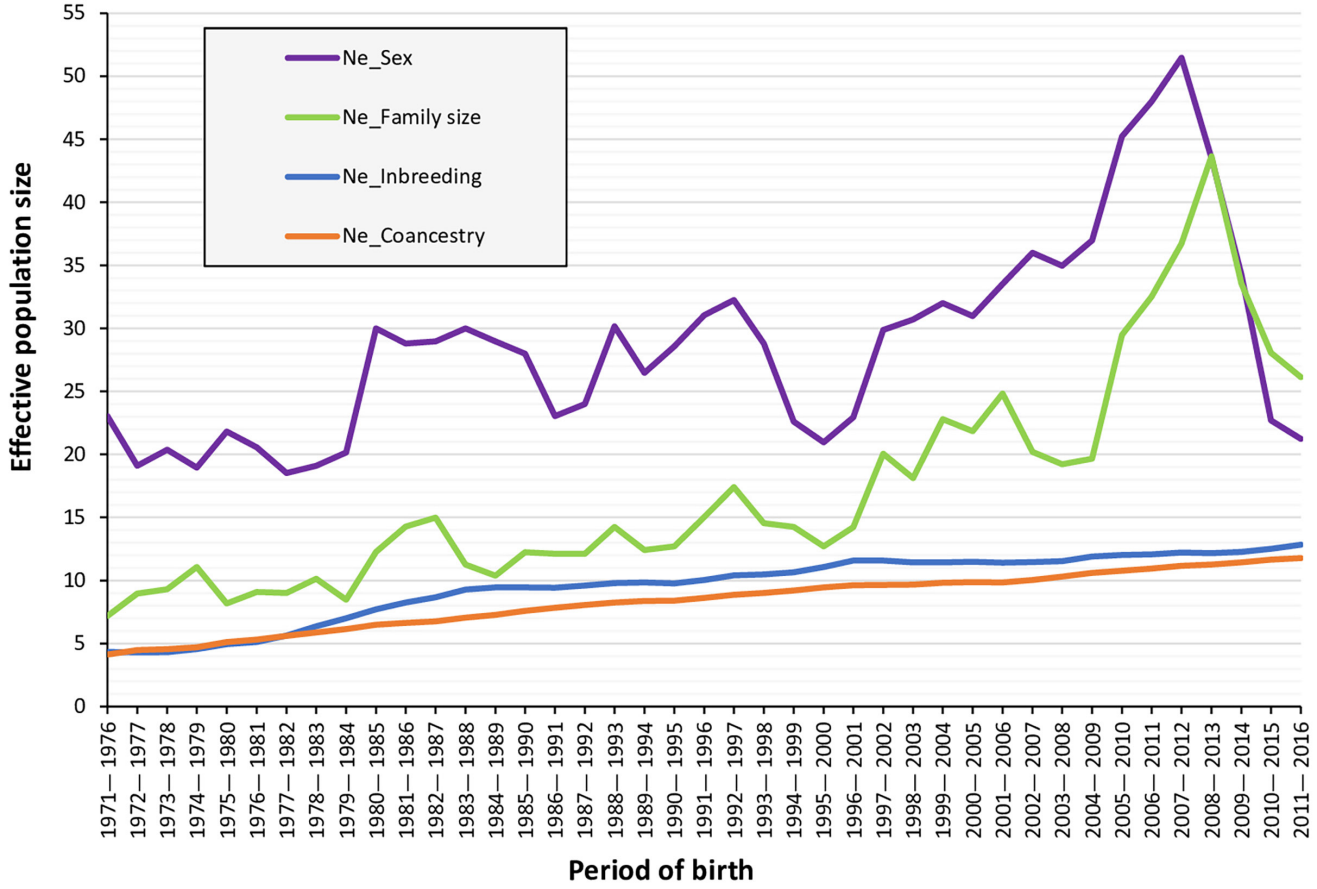
Evolution of the effective population size based on sex ratio, variance of family size, individual increase in inbreeding and individual increase in coancestry by periods of birth in the reference population of Almeria (RPA).

Effective population size values were also calculated using demographic methods (sex ratio and variance of family size) for the RPA (Figure 7). The effective population size values obtained by these methods were more than twice the values calculated from pedigree information, especially for the analysis accounting for only the number of breeding males and females.

### 3.5 *F*‐statistics

**FIGURE 8:**
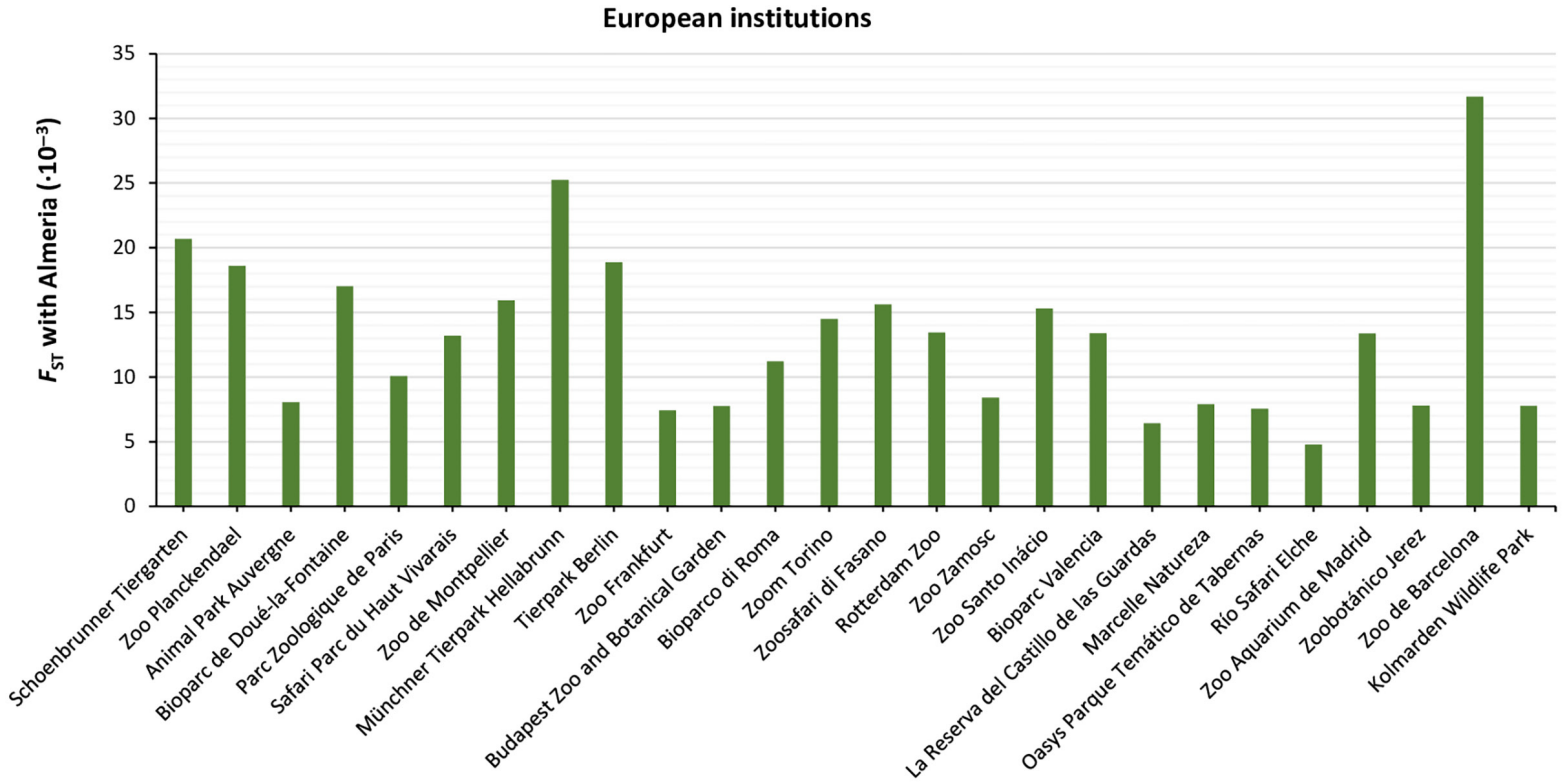
*F*_ST_ distances between Almeria and the other European subpopulations computed from the genealogical information contained in the mhorr gazelle studbook.

Looking at the genetic structure in more detail, the *F*‐statistics computed for the living European population of mhorr gazelle included in the studbook were *F*_IS_ = −0.021377, *F*_ST_ = 0.033370 and *F*_IT_ = 0.012706. *F*_ST_ coefficients between Almeria and the other living subpopulations of Europe were very low (Figure 8), which indicates that there is a close genealogical distance between them.

## 4 DISCUSSION

Management of the captive mhorr population performed over time has resulted in a successful increase in its census, with the number of males born and registered in the studbook being very similar to the number of females. This result indicates that in this population the overall sex ratio at birth did not differ from unity, as found by Moreno et al. (2011) at the age of 30 days, once the period of life with the highest mortality rate for this species is over (Alados & Escós, 1991; Barbosa & Espeso, 2005).

The proportion of animals that have left offspring in the population is not very high, which is much lower in the case of males (16.95% and 41.81%, for males and females respectively). This difference could, however, be explained. While the reproduction of females has been promoted throughout their entire lives along the history of this captive population, management limitations (mainly related to space availability and transport costs between institutions) have prevented all males from being selected for breeding. Although equalizing family size (all individuals in the population producing a similar number of offspring) is often difficult and expensive, it is strongly recommended (Allendorf, 1993) to delay adaptation into captivity (in large captive populations) or when genetic drift is of major concern (in small populations).

The longer average generation interval in the sire‐offspring pathways may be due in part to the high reproductive age of males. In the RPA, it is especially noticeable that the value of the pathway sire‐son is greater than the average value. However, these results should be taken with caution, since the number of data in the RPA and the RPE is limited to draw generalizable conclusions.

The high pedigree completeness level of the mhorr gazelle studbook, higher than in other wild (Armstrong et al., 2011; Skotarczak et al., 2018) and even domestic ruminant species (Gutiérrez et al., 2003; Oliveira et al., 2016), makes it a valuable data set for assessing both, the genetic variability of the mhorr gazelle captive population (Boichard et al., 1997) and its evolution across generations. It is well known that the use of additional molecular genetic techniques helps to clarify, correct and complete the lack of information existing in the pedigree (Jones et al., 2002), in addition to describing the genome‐wide diversity of populations (Steiner et al., 2013). However, despite the recent decrease in sequencing costs (Goodwin et al., 2016) the huge amount of individuals included in the pedigree of the mhorr gazelle makes the use of these techniques unaffordable at the moment of the study. Complete and accurate pedigrees have been shown to explain more variation in inbreeding than microsatellites (Nietlisbach et al., 2017) and provide similar estimates to thousands of SNPs (Galla et al., 2020), making them still a relevant tool in conservation genetics, even more powerful if combined with molecular data. While molecular techniques can help overcome pedigree challenges (e.g. founder relatedness, missing data), robust pedigrees allow for more efficient and economical research work by directing sampling strategies and avoiding massive genotyping (Galla et al., 2022; Perdomo‐González et al., 2022). In addition, pedigrees integrate important metadata related to genealogy that genomic data alone cannot, such as long‐term demographic and life history information (e.g. date of birth, sex, causes of death), or phenotypic traits (e.g. morphometrics, disease susceptibility, reproductive success).

Despite 19 mhorr gazelles (five males and 14 females) arrived at Almeria from the military barracks in the former Spanish colony of Western Sahara between 1971 and 1975 (Cano, 1988; Valverde, 2004), just five of them are considered as unrelated and wild in the current studbook (Domínguez, 2022; see Cano, 1988; Barbosa & Espeso, 2005; Abáigar, 2018 for historical details). However, only four of these five animals (one male and three females) contributed successfully to the perpetuation of the population.

In the three study populations, the ƒ_e_ and ƒ_a_ were less than their respective number of founders and ancestors, which indicates an unbalanced contribution of the founders to the gene pool of the population. The similarities between the ƒ_e_ and ƒ_a_ reflect the absence of new bottlenecks in the history of the captive population registered in the studbook since the beginning of the ex situ breeding program. However, the lower number of founder genome equivalents (ƒ_g_) calculated for the living populations of Almeria and Europe (1.64 and 1.69, respectively) shows that there was a loss of variability due to genetic drift. This value was 1.99 for the whole pedigree, slightly higher than in the reference populations, because in this case the historical coancestries of dead animals were included in the analysis. Consequently, the genetic diversity remaining since foundation (ƒ_g_/ƒ_e_) was 55% in the RPA, 56% in the RPE and 66% in the WP. The results obtained in this study were quite similar to those previously described for the same subspecies by Quilicot and Baumung (2016), where ƒ_e_ = 3.42, ƒ_a_ = 3 and ƒ_g_ = 1.44.

The mean inbreeding coefficients computed here for the WP (*F* = 28.34%), the RPA (*F* = 28.32%) and the RPE (*F* = 28.54%) were higher than some of the first published data (*F* = 11.50% and *F* = 10.05%) by Cassinello (2005) and Ruiz‐López et al. (2009), respectively. However, subsequent studies that considered animals arriving at Almeria being related founders, as currently assumed in the studbook of the species, showed inbreeding coefficient values closer to ours: *F* = 30.10% by Ruiz‐Lopez et al. (2010), *F* = 30.10% by Ruiz‐López et al. (2012) and *F* = 29.71% by Quilicot and Baumung (2016).

As expected from a population with so few founders, the mean average relatedness coefficient is very high in the mhorr gazelle WP (AR = 50.14%). This high value is not common in other captive populations of wild ungulates, such as dorcas gazelles (AR = 11.49%; Veiga, 2016) and European bison (AR = 27.90%; Machová et al., 2022), populations with 24 and 17 founders, respectively. Accordingly, a captive population of addax, descendant of even fewer founders (one male and one female) showed an AR value of 59.50% (Armstrong et al., 2011).

The partial inbreeding coefficients show in more detail the unequal contribution of the four ancestors and its evolution. The presence of a single male made it inevitable that the contribution of this animal is much greater than the contribution of the three females together. In effect, this male is represented in all the descendants. This unequal contribution of the founders, even between the three females, results in a loss of genetic variability in the descendant population (Lacy, 1989). Since no more mhorr gazelles were seen in the wild after 1968, it was not possible to incorporate new founders into the captive population to increase its genetic variability. Another option described to deal with this problem is to enhance the reproduction in captivity of animals poorly represented in the population, in order to balance the contributions of the founders and avoid the loss of alleles by genetic drift (Ballou & Lacy, 1995). Equalizing founder and ancestor representation is generally recommended due to its positive effect on maintaining lower level of inbreeding and higher genetic variation (Loebel et al., 1992). The change in the balance of contributions that has occurred in the mhorr gazelle population can be seen through the partial inbreeding coefficients. As a result of mating control to avoid inbreeding, the representation in the offspring population of the three females with the lowest genetic contribution (ancestors' number 3, 4 and 20) has been increased, while the representation of the only male with the highest genetic contribution (ancestor 1) has been reduced.

The progressive increase over time of $\bar{N_{\mathrm{ei}}}$ and $\bar{N_{\mathrm{ec}}}$ can be seen in both RPA and RPE, indicating that the risk of loss of genetic variability due to genetic drift is decreasing over time in these two reference populations (Vucetich et al., 1997). Furthermore, the comparison between both parameters enables us to identify the mating system carried out in the population (Cervantes et al., 2011). Thus, for the RPA, the values of $\bar{N_{\mathrm{ei}}}$ and $\bar{N_{\mathrm{ec}}}$ are very similar and overlap each other up to year 1977, suggesting a random mating of the population. From 1978 to 2021 the values of $\bar{N_{\mathrm{ei}}}$ and $\bar{N_{\mathrm{ec}}}$ are clearly differentiated and do not overlap each other, as a consequence of the mating selection, based on the minimum coancestry between males and females. This change in the mating system can also be observed in the RPE from 2010. Besides, the ratio $\bar{N_{\mathrm{ec}}}/\bar{N_{\mathrm{ei}}}$ for the RPA (0.92) and for the RPE (0.96) shows how the subdivision of these two populations has been avoided through the mating system. These ratio values below 1 can be observed in populations where coancestry in mating is minimized, as in this case. However, when this ratio was calculated for both reference populations together, the result was 1.04. Although this value is also close to 1, it is slightly higher than the values obtained from the reference populations separately, which could indicate a very small subdivision between the two reference populations. This population structure was possible due to the transfer of a sufficient number of animals between Almeria and European institutions, although, in some cases, sanitary restrictions and geographical distances have prevented the exchange of animals between both reference populations from being totally free. This consequence is most notable in the WP (ratio 1.12), where limitations on the exchange of specimens between continents to achieve optimal matings could have caused a slight subdivision within the global population.

The notable difference in the *N*_e_ value depending on the calculation method used makes the results obtained in this investigation from pedigree information difficult to compare with other values based on the sex ratio previously reported by Cassinello in 2005 (*N*_e_ = 7) and Ruiz‐López et al. in 2009 (*N*_e_ = 6.55). This demographic method is also used to estimate the minimum viable population sizes established by the 50/500 rule, being these numbers the proposed thresholds of the *N*_e_ value for the risk of extinction in the short and long term, respectively (Franklin, 1980). In a similar way to the *N*_e_ computation, the Food and Agriculture Organization (FAO) based its recommendations on sex ratio considerations to determine the level of endangerment (Rischkowsky & Pilling, 2007). Consequently, these demographic methods have been criticized for not providing a full picture of the level of genetic diversity (Leroy et al., 2013; Martyniuk et al., 2010). However, the need for a simpler global scale and limitations of pedigree information on certain occasions (i.e. for wild populations) meant that demographic methods were selected as criteria. Therefore, although the proposed thresholds remain useful as rough measures for comparing populations when only the number of breeding animals is known, their limitations must be well known in order to avoid incorrect management decisions (Harmon & Braude, 2010). The inclusion of the *N*_e_ and other genetic parameters as criteria for classifying species within the IUCN Red List of Threatened Species has been proposed (Garner et al., 2020; Willoughby et al., 2015), in addition to the demographic criteria that are already taken into account, such as the decrease in population size, the extension of the range of species and the number of mature individuals. Since genetic diversity is important to ensure the survival of species (Frankham, 1996), its loss should be considered as a risk for the disappearance of populations, as well as natural systems modifications or problems with invasive species, among others. The present study is an example of the usefulness of *N*_e_ to expand knowledge about the genetic variability of a threatened population and its evolution over time, as well as the structure of the population and the mating strategies implemented in the captive breeding programs.

According to the value of *F*_IT_, the inbreeding of the living European population is slightly increasing in the EEP of the mhorr gazelle, but the negative genealogical *F*_IS_ value means that mating between relatives is being avoided (Toro et al., 2000). *F*_ST_ value computed for the living European population (*F*_ST_ = 0.033370) suggests a very small level of differentiation between subpopulations. It is due to the common origin of all subpopulations, descendants of the same wild ancestors brought from Africa to Almeria, and the subsequent exchange of individuals within the captive breeding program. In addition, the paired *F*_ST_ distances obtained between the different living subpopulations of Europe and Almeria were low. Río Safari Elche was the most closely related subpopulation to Almeria. This is explained because all the individuals from this institution come directly from the population of Almeria in year 2021. On the opposite side, Zoo de Barcelona was the least related, possibly because its genetic exchange with Almeria has been less than with other European subpopulations (only eight animals in 1994 and 1998). To have information on the value of these genetic distances between subpopulations is useful in programming matings within the breeding program, helping to promote reproduction among less related subpopulations, to the extent that other kind of restrictions allow it (for example, geographical distance).

In summary, we can say that despite the low number of founders that originated the ex situ conservation program of the mhorr gazelle, the *N*_e_ value has been progressively enhancing, showing how the increase in the census and the mating management allow maximizing the maintenance of the genetic variability in this population. Selection of minimal coancestry matings has also prevented the recurrence of new bottlenecks and big subdivisions in the population. This may be due to the genetic purging process recently described in the mhorr gazelle population by López‐Cortegano et al. (2021), a process by which alleles are removed by selection, thus improving some fitness traits over generations and reducing inbreeding depression (Lande & Schemske, 1985). The retention of enough genetic variability, maximizing the genetic representation of all the wild‐caught founders, is essential for future adaptation, successful expansion and restoration of natural populations (Frankham, 1996; Hedrick & Miller, 1992). The fact that reintroduced mhorr gazelle populations have not been able to prosper enough in the long term could indicate a lack of evolutionary potential or the magnification of inbreeding depression in stressful natural environments, such as changes in the habitat, less food availability or the presence of predators (Abáigar, 2018; Abáigar et al., 2019; Cano et al., 1993; Moreno et al., 2012). To determine the adaptive potential of this captive population, more studies on its fitness are necessary. This would allow identifying individuals that have better reproductive and survival traits to be released into the wild with greater possibilities of success.

## AUTHOR CONTRIBUTIONS

**Sonia Domínguez:** Conceptualization (equal); data curation (lead); formal analysis (lead); writing – original draft (lead); writing – review and editing (equal). **Isabel Cervantes:** Conceptualization (equal); formal analysis (equal); methodology (equal); supervision (equal); writing – review and editing (equal). **Juan Pablo Gutiérrez:** Conceptualization (equal); formal analysis (equal); methodology (equal); software (lead); supervision (equal); writing – review and editing (equal). **Eulalia Moreno:** Conceptualization (equal); formal analysis (supporting); supervision (equal); writing – review and editing (equal).

## CONFLICT OF INTEREST STATEMENT

None declared.

## Supporting information


Appendix S1.

## Data Availability

Pedigree information uploaded as online: http://www.eeza.csic.es/documentos/Studbook_2021_Nanger_dama_mhorr.pdf.
