# Supplementary material for: Pedigree analysis in the mhorr gazelle (Nanger dama mhorr): Genetic variability evolution of the captive population
Source: Ecol Evol. 2024 Feb 17;14(2):e10876. doi: 10.1002/ece3.10876 (PMC10873689; doi:10.1002/ece3.10876)
Supplement: Supplementary file 1 — Appendix S1. [file ECE3-14-e10876-s001.docx]

**SUPPLEMENTARY MATERIAL**

**APPENDIX S1.** Name, acronym and country of all the institutions included in the mhorr gazelle studbook (updated at 31 December 2021). The complete studbook can be found here: <http://www.eeza.csic.es/documentos/Studbook_2021_Nanger_dama_mhorr.pdf>

| Studbook acronym | Institution name | Country |
| --- | --- | --- |
| AFRICAN | Unknown | Unknown |
| AGADIR | Unknown | Morocco |
| AL AIN | Al Ain Zoo | United Arab Emirates |
| AL AREEN | Al Areen Wildlife Park | Bahrain |
| ALBG | Al Bustan Gazelle Farm | United Arab Emirates |
| ALBUSTAN | Al Bustan Zoological Centre | United Arab Emirates |
| ALMER.ICO | Instituto para la Conservación de la Naturaleza | Spain |
| ALMERIA | Estación Experimental de Zonas Áridas | Spain |
| AUVERGNE | Animal Park Auvergne | France |
| BARCELONA | Zoo de Barcelona | Spain |
| BELFAST | Belfast Zoological Gardens | United Kingdom |
| BERLIN TP | Tierpark Berlin | Germany |
| BIOPARCVA | Bioparc Valencia | Spain |
| BODE W | Werner Bode | Germany |
| BOU HEDMA | Bou Hedma National Park | Tunisia |
| BOWMANVIL | Bowmanville Zoological Park | Canada |
| BREMEN | Bremer Tierpark | Germany |
| BREMEN QU | Wormer II Quarantine Ship | Germany |
| BUDAPEST | Budapest Zoo and Botanical Garden | Hungary |
| BUSCH TAM | Busch Gardens Tampa Bay | United States |
| CABARCENO | Parque de la Naturaleza de Cabárceno | Spain |
| CINCINNAT | Cincinnati Zoo and Botanical Garden | United States |
| CORDOBA | Parque Zoológico de Cordoba | Spain |
| DISNEY AK | Disney's Animal Kingdom | United States |
| ELCHE SAF | Río Safari Elche | Spain |
| ESTEPONA | Selwo Aventura | Spain |
| FASANO | Zoosafari de Fasano | Italy |
| FONTAINE | Bioparc de Doué-la-Fontaine | France |
| FRANKFURT | Zoo Frankfurt | Germany |
| HLUBOKA | Zoo Hluboká | Czechia |
| HOLIDAY | Earl Tatum | United States |
| INDETERMI | Indeterminate location | Unknown |
| IZS | International Zoo Services | Germany |
| JEREZ | Zoobotánico Jerez | Spain |
| JOHNSON L | Safari Enterprises (Larry Johnson) | United States |
| KOLMARDEN | Kolmarden Wildlife Park | Sweden |
| LANGLEY | Mountain View Conservation and Breeding Centre | Canada |
| LOUISVILL | Louisville Zoo | United States |
| MADRID Z | Zoo Aquarium de Madrid | Spain |
| MARATHON | Iron Mountain Ranch (Brad Kelley) | United States |
| MARCELLE | Marcelle Natureza | Spain |
| METROZOO | Zoo Miami | United States |
| MNC UAE | Management of Nature Conservation | United Arab Emirates |
| MONTPELLI | Zoo de Montpellier | France |
| MUNICH | Münchner Tierpark Hellabrunn | Germany |
| NASHV ZOO | Nashville Zoo at Grassmere | United States |
| NURNBERG | Tiergarten Nürnberg | Germany |
| OKLAHOMA | Oklahoma City Zoo and Botanical Garden | United States |
| OR WILDLF | Oregon Wildlife Foundation | United States |
| OSNABRUCK | Zoo Osnabrück | Germany |
| PALM DES | The Living Desert Zoo and Gardens | United States |
| PARIS ZOO | Parc Zoologique de Paris | France |
| PEACE RV | Peace River Wildlife Center | United States |
| PEAUGRES | Safari Parc du Haut Vivarais | France |
| PHILADELP | Philadelphia Zoo | United States |
| PHOENIX | Phoenix Zoo | United States |
| PLANCKNDL | Zoo Planckendael | Belgium |
| PRET LICH | Lichtenburg Game Breeding Center | South Africa |
| PRETORIA | National Zoological Gardens of South Africa | South Africa |
| QUINTASI | Zoo Santo Inácio | Portugal |
| RABAT | Jardin Zoologique National de Rabat | Morocco |
| R'MILA | R'Mila Royal Reserve | Morocco |
| ROMA | Bioparco di Roma | Italy |
| ROTTERDAM | Rotterdam Zoo | Netherlands |
| SANDIEGOZ | San Diego Zoo | United States |
| SD-WAP | San Diego Zoo Safari Park | United States |
| SENEGAL | Réserve Spéciale de Faune de Guembeul | Senegal |
| SEVILL RN | La Reserva del Castillo de las Guardas | Spain |
| SOUS MASS | Souss Massa National Park | Morocco |
| ST LOUIS | Saint Louis Zoological Park | United States |
| TABERNAS | Oasys Parque Temático de Tabernas | Spain |
| TWYCROSS | Twycross Zoo | United Kingdom |
| UNDETERMI | Undetermined location | Unknown |
| UNKNOWN | Unknown location | Unknown |
| VERGEL PK | Safari Park Vergel | Spain |
| VIENNA | Schönbrunner Tiergarten | Austria |
| W.AFRICA | Wild | Morocco |
| WOBURNLTD | Woburn Safari Park | United Kingdom |
| ZAMOSCZSM | Zoo Zamosc | Poland |
| ZOOMTORIN | Zoom Torino | Italy |
